# Biographical Memoirs of Dr. Pitcairn

**Published:** 1809-06

**Authors:** 


### *Biographical Memoirs of Dr. Pitcairn.*

DAVID PITCAIRN was the eldest son of the gallant Major John Pitcairn, of the Marines, who was killed in the attack on Bunker's Hill in June 1775, and Elizabeth, the daughter of Robert Dalrymple, Esq. of Annefield, in the County of Dumfries. His paternal family was one of the most ancient in Fifeshire, deriving its name from a landed possession called Pitcairn; Nisbett in his Heraldry says, that he has seen a charter to it dated in 1417. In the course of time one of the family acquired by marriage the estate of Forther, in the same county; after which the lands of Pitcairn went off with a younger son, from whom was descended Dr. Archibald Pitcairn, of Pitcairn, justly famed as a physician, poet, wit, scholar, and mathematician. Of the elder branch, Dr. David Pitcairn became the representative upon the death of his uncle, the well-known Dr. William Pitcairn, who had practised physic in London for nearly half a century, and had been many years President to the College of Physicians.

David was born on the 1st of May, 1749, in the house of his grandfather, the Rev. David Pitcairn, minister of Dysart, in the County of Fife. When about nine or ten years old, he was sent to the High School at Edinburgh, where he remained four years; after which he went to the University of Glasgow, and prosecuted his studies there till he arrived at the age of twenty. At this period of his life he used to spend much of his leisure time with the family of the Rev. James Baillie, minister of Bothwell, in the County of Lanark, and the father of the present Dr. Mathew Baillie, of London, and of the celebrated dramatic writer Miss Johanna Baillie. During this intercourse commenced an affectionate intimacy between Dr. Pitcairn and Dr. Baillie, which afterwards, as the difference of their years became less in proportion to their whole ages, gradually changed into the warmest friendship, that continued ever after. In 1769, David went, however, to the University of Edinburgh, and studied medicine there for three years, under the immediate direction of the illustrious Cullen. In 1772 he came to London, and attended the lectures of his uncle's learned friends, Drs. W. Hunter and G. Fordyce. About the same time also, that he might attain an English degree in Physic, being then nearly 23 years old, he entered at Bennet College, Cambridge. In 1780, several years before he received his

Doctor's Degree, he was elected Physician to St. Bartholomew's Hospital, and in 1783, Fellow of the Royal Society. He was soon afterwards appointed Physician to the Prince of Wales, and about the same time may be considered the commencement of his private medical practice. In 1792 he was chosen Physician to Christ's Hospital; and in the following year, his private practice being now considerable, he resigned the office of Physician to St. Bartholomew's Hospital. His office at Christ's Hospital requiring but little of his time, was retained by him several years longer.

By the death of Dr. Warren, which took place in June 1797, Dr. Pitcairn was placed at the head of his profession in London. One or two other physicians possibly derived as much pecuniary emolument from the practice of medicine as himself; but certainly no other one was so frequently consulted by his brethren in cases of difficulty. But this prosperous state did not endure long. In the autumn of the same year, by a fall from his horse, he bruised his side. Shortly after, his heart began to beat with violence. His attention was more particularly directed to this symptom, as it had occurred from a similar accident in one of his brothers, whose heart, after death, was found considerably enlarged. He continued, however, to follow his profession till February in the following year, when he was attacked with a hæmorrhage from his lungs. From this he recovered, after some time, so far as to be enabled to resume the exercise of his profession; but the same disease having recurred in summer, he embarked in September for Lisbon. During a stay of more than 18 months in Portugal, he had no return of the hæmorrhage, in consequence of which he ventured to return to this Country in May 1800. He was still, however, feeble, and his heart still beating too forcibly, he, for some time longer, declined altogether engaging in medical practice. As his health improved, he began to receive patients at his house; then to meet other physicians in consultation at the houses of their patients; and at length, after an interval of several years, to undertake business in the usual way, except during four months in the latter part of the year, which he spent principally in the country. In the mean time, however, the palpitation of his heart continued; on which account for a long time he lived very abstemiously, drinking only water, and abstaining almost entirely from animal food. But, the palpitation not increasing, and no other sign of a diseased heart appearing, as he found a vegetable diet to produce much flatulence, about a year or two before his death he began to eat moderately of animal food once a day, and to take sometimes after dinner a single glass of wine diluted with water. Under this change of regimen his appearance altered considerably, and during the last six months of his life he frequently received the congratulations of his friends on the improvement which his health had undergone. The danger of high health in a constitution disposed to hæmorrhage, and probably to plethora, would not have been overlooked by so sagacious a physician in any subject but himself, or perhaps, he considered that by hard exercise the danger might be obviated. In the course of the month of March, he rose several times from his bed soon after midnight, and travelled between twenty and thirty miles before morning, to visit a patient.

From these exertions he appeared to suffer no immediate injury. But about the beginning of April finding himself heated by his single glass of wine, though diluted largely with water, he discontinued it. On the 12th he felt a soreness in his throat; but thought so slightly of it, that he continued his professional visits during that and the two following days. In the night of the 15th, his throat became worse, in consequence of which he was copiously bled at his own desire, and had a large blister applied over the throat; but the irritation occasioned by the latter remedy was so distressing to him, that it was removed before its intended effect was fully produced. On the evening of the 16th Dr. Baillie called upon him, without knowing that he was ill; and having heard the history of his ailment, and an account of the remedies employed, he entirely approved of what had been done. At this time there appeared no symptom which indicated danger. The disease becoming more violent in the course of the night, a considerable number of leeches were applied to the throat early in the morning. Dr. Baillie visited him at 11 o'clock in the forenoon. His countenance was now sunk, his pulse feeble and unequal, his breathing laborious, and his voice almost lost, from the swollen state of the organs concerned in its formation. In this state he wrote upon a piece of paper, that he conceived the trachea to be the principal seat of the disease, and that this was the Croup. Mr. Home was also present; and it was agreed that an attempt should be made to give relief by wounding the tonsils. This was accordingly done; some blood issued, but nothing purulent. Both the patient, however, and those about him, conceived that he had derived benefit from the operation. Dr. Baillie saw him again between four and five o'clock in the afternoon, and thought his situation much improved; for the pulse was now equal and more firm, and his general appearance indicated less debility and distress. Under this persuasion he left him, having previously agreed to return at 10 in the evening, when he was to meet in consultation Mr. Home, and another physician who had long been intimate with his patient. A little before Dr. Baillie had paid the visit just mentioned, a slight drowsiness had come on, and this symptom rather increased after his departure. But nothing more remarkable occurred till near eight o'clock, when the patient's breathing became suddenly more difficult. About 20 minutes after this he died.

The body was examined the second day after death by Mr. Home; The tongue was much swollen and inflamed, the under part of the tip being exceedingly red. The glands at the root of the tongue unusually conspicuous. The epiglottis was inflamed and extremely thick, the internal membrane of the larynx was inflamed, and the orifices of the sacculi laryngei were swelled, and an appearance not unlike matter in them. The same inflammation was seen upon the internal membrane of the trachæa. The heart and lungs were in a healthy state.

On the 25th, his corpse was deposited in a vault in the Church of St. Bartholomew, near Smithfield, which contained the remains of his father and uncle.

Dr. D. Pitcairn had five brothers; one of them died young; three others, all of them officers in his Majesty's service, died after they were men; the youngest, a Counsellor at Law, survives him. He had four sisters, all of whom have been married, and are alive. His mother also still lives, and is in her 79th year. In 1781, he married Elizabeth the only daughter of William Almack, Esq. of London, and a niece of his preceptor, Dr. Cullen, but had no issue. She likewise survives him.

His person was tall and erect, but of late years rather thin, till he recovered health; his countenance during youth was a model of manly beauty, and even in advanced life was remarkably handsome. While a boy, he was noted for possessing a grave and manly manner, connected with much sweetness of disposition. These qualities, added to considerable bodily strength and courage, gave him great influence over his play-fellows. He did not acquire knowledge at school as quickly as some of his companions, yet yet as his memory, however, was strong, and his judgment sound, whatever he learned he retained and assorted, so that in time he excelled most of those who had once been regarded his superiors. His knowledge of History and Geography, from the strength of his memory, was particularly accurate.

Few persons ever gained, without any direct effort to this end, so extensive an acquaintance with the various orders of society. His education began at the largest school in Great Britain. He afterwards studied for several years at each of the great Universities of Glasgow, Edinburgh, and Cambridge, and attended the principal Lectures upon Medicine in London. While a young man he lived in London with his uncle, who had many friends, and frequently entertained them at his house. He resided many years in Lincoln's Inn Fields; and, while there, associated daily with Gentlemen of the Law. He was early admitted a Fellow of the Royal and Antiquarian Societies; and hence knew many learned men, in addition to those of his own profession. He was fond of country sports and athletic games, particularly the Scottish one named Golf, which carried him among other sets of men. He had a taste also for the Fine Arts; in consequence of which, he became acquainted with many of the professors of them; and his employment as a Physician in the largest hospital in the metropolis, and in private, made known to him a very great number of persons of every rank and description in life. From such opportunities, and an original turn for the observation of character, he obtained a most extensive knowledge of human nature, and an infinite fund of stories and anecdotes, which, when at ease among his friends, he used to relate in the happiest way. None of his stories, however, related to himself; indeed, he scarcely ever spoke of himself to his most intimate friends; no doubt, from a wish to avoid a fault he saw so frequently committed by others. In conversation he shunned dispute. When he dissented from others, he either declared his opinion in a few words, or remained altogether silent. With literary men his value as a companion was considerably increased by his judgment in selecting, and lively mode of repeating, passages from new works of taste, most of which he read immediately after they were published. But, though he had lived so much in society, he never entirely lost a natural shyness of manner, which was more observable at some times than at others. This was sometimes imputed by those who did not know him to pride; though nothing was further from his real character. As he advanced in years, his manners became less reserved to strangers; for, to his friends, they had always been frank and affectionate.

His feelings were so warm as sometimes to betray him into little improprieties; but this disadvantage was greatly outweighed by the energy which was hence given to his character, and the interest which he took in the happiness of others. It may be regarded, perhaps, as no considerable title to praise, that he behaved with the utmost kindness and generosity towards his numerous relations. But his endeavours to serve were not confined to these. He was ever ready to assist his friends in their pursuits, by his advice, and still more by his influence with others, and the sacrifice of his time; to say nothing of other aids which he frequently furnished. Like other men of warm tempers, he was apt to bestow upon his present pursuits more than their due importance; and, as increase of years and professional employments, together with great varieties in the state of his health, necessarily produced alterations in his views of life, he was hence thought by some to be of a changeable disposition. But this was never said, respecting his attachment to persons. He continued to the last, loving his first friends, and was, in return, most cordially beloved by them.

His manner, as a physician, was simple, gentle, dignified, and always sufficiently chearful to encourage hope, without offending by its incongruity with the scene about him. From his kindness of heart, he was frequently led to give more attention to his patients than could well be demanded from a Physician; and as this evidently sprung from no interested motive, he often acquired considerable influence with those whom he had attended during sickness. No Physician indeed of his rank in London, perhaps, ever exercised his profession to such a degree gratuitously. His behaviour to other physicians was highly candid and liberal, and he most studiously avoided the slightest appearance of interfering in their professional concerns. Such conduct is, no doubt, recommended by its ultimate utility; but in him it was only a part of his native dignity of character.

As he attended very carefully to the symptoms of diseases, in the order and degree in which they occur in nature, he had, from this source, and the excellence of his memory, acquired great practical knowledge of his profession. He had, in consequence, also made many original ginal observations upon the history and treatment of diseases. He never published any thing himself, but several of his observations have been given to the world by others. When he was some years ago urged to publish, his answer was, that he did not wish seven years hence to be obliged to contradict his present opinions.

He never long enjoyed very good health from the time of his commencing to practise Physic in London. For, besides what has already been said respecting his disorders, he was, during many years of the first part of his residence here, much subject to violent head-achs. He twice laboured under severe agues; and suffered several attacks of inflammatory sore-throat. None of his ailments made any considerable permanent impression upon his external appearanee; for, immediately before his death, no person would have supposed that his health had ever failed, or that he had arrived at nearly his sixtieth year.

